# Belimumab in early systemic lupus erythematosus: A propensity score matching analysis

**DOI:** 10.1002/iid3.1362

**Published:** 2024-08-22

**Authors:** Chaofan Lu, Nan He, Lei Dou, Hongxia Yu, Mengtao Li, Xiaomei Leng, Xiaofeng Zeng

**Affiliations:** ^1^ Key Laboratory of Rheumatology and Clinical Immunology, Department of Rheumatology and Clinical Immunology, National Clinical Research Center for Dermatologic and Immunologic Diseases (NCRC‐DID), Peking Union Medical College Hospital, Chinese Academy of Medical Sciences & Peking Union Medical College Ministry of Education Beijing China; ^2^ Department of Rheumatology, Sir Run Run Shaw Hospital Zhejiang University School of Medicine Hangzhou China; ^3^ Department of Rheumatology and immunology The Second People's Hospital of Wuhu Wuhu China; ^4^ Department of rheumatology Guizhou Xingyi people's Hospital Xingyi China

**Keywords:** belimumab, early medical intervention, LLDAS, lupus nephritis, systemic lupus erythematosus

## Abstract

**Objective:**

This study aimed to evaluate the clinical efficacy of belimumab in patients with early systemic lupus erythematosus (SLE), defined as having a disease duration of less than 6 months.

**Methods:**

We retrospectively identified patients with SLE in the early stage who received belimumab and standard of care (belimumab group) or standard of care alone (control group) since September 2020. Propensity score matching (PSM) was used to reduce potential bias. The primary endpoint was lupus low disease activity status (LLDAS) at weeks 12 and 24. The secondary endpoints were remission and the proportion of glucocorticoid dose tapering to 7.5 mg/day. The efficacy of belimumab in patients with lupus nephritis was also assessed.

**Results:**

Out of 111 eligible patients, 16 patients in the belimumab group and 31 patients in the control group were identified by 1:2 PSM. At week 24, a significantly higher proportion of individuals achieved low disease activity state (LLDAS) in the belimumab group compared to the control group (56.3% vs. 19.4%, OR = 5.357, 95% CI = 1.417 to 20.260, *p* = 0.013). Furthermore, more patients in the belimumab group were reduced to low‐dose glucocorticoid ( ≤ 7.5 mg/day) at week 24 (75.0% vs. 35.5%, OR = 5.182, 95%CI = 1.339 to 20.058, *p* = 0.017). Significant improvements in Patient Global Assessment scores were observed at Week 12 and 24 for those treated with belimumab compared to controls. In a subgroup analysis evaluating the efficacy of belimumab in patients with lupus nephritis, 42.9% of the seven individuals treated with belimumab achieved a complete renal response (CRR) by Week 24, and no instances of disease relapse were observed.

**Conclusions:**

In SLE patients with a disease duration of less than 6 months, belimumab treatment can promote LLDAS achievement and reduce glucocorticoid dose, leading to a better prognosis. Introducing belimumab in the early stage of SLE may be a beneficial decision.

## Graphical abstract

A retrospective case‐control study with propensity score matching to measure the efficacy of belimumab in individuals with early systemic lupus erythematosus (SLE) (disease duration of less than 6 months). Our study suggests that belimumab contributed to reaching LLDAS status and reducing GCs doses in SLE patients with a disease duration of fewer than 6 months.

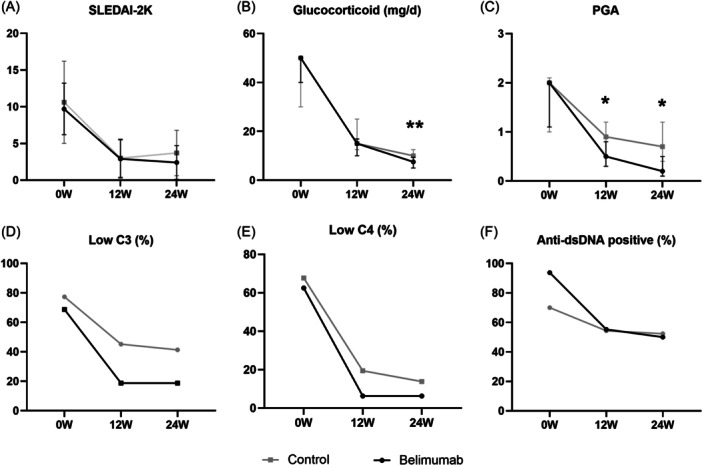

## 1 INTRODUCTION

Systemic lupus erythematosus (SLE) is a chronic diffuse connective tissue disease with an unknown etiology, characterized by diverse clinical manifestations and involvement of multiple organ systems. Loss of B‐cell tolerance and over‐activation of T‐cells are believed to contribute to the pathogenesis of SLE.^1^ Despite the improved survival rates observed in SLE patients over the past five decades,^2^, ^3^ significant challenges exist in the diagnosis and clinical management of this disorder, including residual disease activity, frequent flares, glucocorticoid (GC) reliance and associated toxicity, and organ damage.^4^ Several studies found that when the lag time between the first symptom to the diagnosis of SLE was less than 6 months, patients had a lower risk for flares, hospitalizations, healthcare utilization, and organ damage, although there is no universally accepted definition of early SLE.^5^, ^6^, ^7^

B‐lymphocyte stimulator (BLyS) protein promotes the survival and differentiation of B lymphocytes into immunoglobulin (Ig)‐producing plasma cells, and its role in the pathogenesis of SLE is well‐documented.^8^, ^9^ The anti‐BLyS monoclonal antibody, belimumab, has been approved for the treatment of SLE,^10^, ^11^ and clinical studies and post‐marketing real‐world data attest to the efficacy and safety of belimumab.^12^, ^13^, ^14^ BLISS‐LN study also showed that belimumab plus standard therapies for lupus nephritis enhanced renal responses.^11^ A recent study, BeRLiSS, demonstrated that shorter disease duration and lower baseline organ damage were predictors of disease remission with belimumab treatment.^15^ However, there is limited data on Chinese or Asian patients with SLE at early stage using Belimumab. A single‐center retrospective study provided evidence about efficacy of belimumab in patients recently diagnosed with SLE.^16^ However, this study lacks a control group, which results in a weakened strength of the research conclusions. Therefore, we conducted a retrospective case‐control study with propensity score matching to measure the efficacy of belimumab in individuals with early SLE (disease duration of less than 6 months) and lupus nephritis.

## 2 MATERIALS AND METHODS

### 2.1 Study design and population

This study included patients diagnosed with SLE based on the 1997 SLE classification criteria revised by the American College of Rheumatology (ACR)^17^ or 2012 Systemic Lupus International Collaborating Clinics (SLICC) classification criteria for SLE^18^ with disease duration of fewer than 6 months since September 2020. Disease duration was defined as the duration between the first appearance of any symptom/sign attributed to SLE and baseline. The primary exclusion criteria were exposure to any B‐cell–targeted therapy, excluding belimumab, within a year. Patients who had missing follow‐up data at either Week 12 or Week 24 were also excluded.

The sample size was determined based on response rates from previous studies. Patients treated with Belimumab for 24 weeks had a response rate of 75.2% (BeRLISS study),^15^ while those on standard treatment had rates between 18.8% and 44.2%.^19^, ^20^ We predicted a 35% response rate in the standard treatment group. With a 1:2 ratio for Belimumab and standard treatment, a significance level of α = 0.05 (two‐sided), and power of 0.8, we calculated that 15 cases in the Belimumab group and 30 cases in the standard treatment group were required.

The study population was divided into two groups based on the use of belimumab, namely the belimumab group (belimumab and standard of care) and the control group (standard of care only). Among them, 16 patients were included in the belimumab group while 95 patients were included in the control group for subsequent analysis. Standard of care treatment included the use of GCs, antimalarials, and selective use of immunosuppressants as deemed necessary. This study was approved by the Medical Ethics Committee of Peking Union Medical College Hospital (ZS‐3063). All patients gave their signed informed consent.

### 2.2 Data collection

Data collected for analysis included the following categories: 1) Demographic data, which included age and gender. 2) Clinical data, which included disease duration, the Systemic Lupus Erythematosus Disease Activity Index‐2000 (SLEDAI‐2K),^21^ patient global assessment (PGA; scale 0–3)^22^; and the use and doses of GC and immunosuppressants (IS); The prescribed GC dosage was converted to prednisone equivalent dose; 3) Laboratory results, which included antinuclear antibody (ANA), anti‐double‐stranded DNA (anti‐dsDNA), 24‐h urine total protein (24hUP), complement C3, and C4. The complement levels were assessed using nephelometry, with a lower limit of normal for complement C3 at 0.73 g/L and a lower limit of normal for complement C4 at 0.1 g/L. The presence of anti‐dsDNA was determined through indirect immunofluorescence assay, where a titer of 1:10 or higher is considered indicative of a positive result.

### 2.3 Main outcome measures

The main outcomes were measured at Week 12 and Week 24. The primary efficacy outcome was evaluated based on lupus low disease activity status (LLDAS).^23^ LLDAS was defined as SLEDAI‐2K score≤4, no activity in any major organ, no new disease activity feature, PGA ≤ 1, prednisone ≤7.5 mg/day, and allowance for the continuation of IS and antimalarials.^22^ The major secondary efficacy outcomes were remission, defined as (2021 DORIS): a clinical SLEDAI‐2K of 0, PGA < 0.5, low‐dose GC (prednisolone ≤5 mg/day), and/or stable IS including biologics.^24^ The proportion of patients with glucocorticoid dose tapering from baseline to 7.5 mg/day (converted to prednisone equivalent dose) was also evaluated. Other endpoints included the percentage of patients with at least a 4‐point reduction from baseline in SLEDAI‐2K, mean change in SLEDAI‐2K, mean change in PGA score, percentage of patients with normalization of complement, and percentage of patients with the conversion from anti‐double stranded DNA (anti‐dsDNA) positive to negative.

Furthermore, we conducted an analysis of the therapeutic response in patients with lupus nephritis (LN) at baseline. In accordance with the criteria in BLISS‐LN,^11^ we defined Complete Renal Response (CRR) as Calculated glomerular filtration rates (GFR) not worse than 10% below pre‐flare value or ≥90 mL/min/1.73 m^2^, and Inactive urinary sediment (<5 red blood cell/high power field and <5 white blood cell/high power field [or within laboratory reference range]), and no cellular casts (no red blood cell and/or white blood cell casts), and uPCR/24hUP < 0.5. Partial Renal Response (PRR): Estimated or calculated GFR not worse than 10% below baseline value or within normal range, and red blood cell/high power field ≥50% reduction from baseline or <5 red blood cell/high power field (or within the laboratory reference range), and no red blood cell casts and decrease in the uPCR/24hUP ≥ 50% with one of the following: uPCR/24hUP < 1.0, if baseline uPCR/24hUP ≤ 3.0; or uPCR/24hUP < 3.0, if baseline ratio >3.0. Lastly, patients who did not meet the above criteria were classified as Nonresponders.

### 2.4 Statistical analysis

In this study, categorical variables were described using frequencies and percentages, while continuous variables were presented as mean (SD) or median (IQR). The normality was tested by the Shapiro‐Wilk test. The association between categorical variables was determined using either Pearson's χ2 test or Fisher's exact test. The student's t‐test or Mann–Whitney U test was used for continuous data as appropriate. Statistical analysis was performed using SPSS 22.0 (IBM). *p* < .05 (2‐sided) was considered statistically significant. Subgroup analysis was conducted according to different clinical populations. To make the two groups comparable at baseline, propensity score matching was performed with SPSS 22.0 embedded with a 1: n propensity score matching (PSM) plug‐in, following the guidelines outlined in Yang et al.'s article.^25^ The propensity score was calculated by using logistic regression analysis, including age, gender, and SLEDAI‐2K scores. Patients in the belimumab group were matched in a 1:2 ratio to those in the control group using nearest neighbor matching with a 0.1 caliper width. The efficacy of belimumab was assessed with consideration given to the use of immunosuppressants and baseline glucocorticoid dosage as covariates with the multivariate logistic regression analysis. Each immunosuppressant's utilization status is treated as a distinct variable and simultaneously integrated into the regression model.

## 3 RESULTS

### 3.1 The baseline characteristics of patients

Before PSM, 16 patients in the belimumab group and 95 patients who received standard of care (SoC) were included. There were significant imbalances (SMD > 0.1) between the two groups in terms of gender and SLEDAI, which are recognized as strong risk factors for the outcome and were addressed through PSM. After PSM, 16 patients in the belimumab group and 31 patients who received SoC were included (Table 1). We observed that there was a balanced gender distribution and similar SLEDAI levels between the two groups following PSM. Furthermore, variables not included in the propensity score model also showed decreased SMDs, indicating an improvement in between‐group differences. While there still existed some disparity in the distribution of mucocutaneous, musculoskeletal, and serositis involvement, major affected organs such as hematological, renal, and central nervous systems exhibited a more balanced pattern. Additionally, discrepancies in the choice of immunosuppressive agents were noted. As a result, the choice of immunosuppressive agents was included as confounding factors in the subsequent multivariate analysis for efficacy testing due to its impact on treatment outcomes.

**Table 1:** Demographic and clinical characteristics of patients at baseline.

	Before PS matching			After PS matching		
	Belimumab (*n* = 16)	Control (*n* = 95)	SMD	Belimumab (*n* = 16)	Control (*n* = 31)	SMD
Gender, female	14 (87.5)	89 (93.7)	0.213	14 (87.5)	28 (90.3)	0.090
Age, years	30.25 (11.70)	31.40 (11.30)	0.100	30.25 (11.70)	30.94 (11.42)	0.059
Disease duration, months	2.19 (1.22)	2.08 (1.55)	0.074	2.19 (1.22)	2.29 (1.53)	0.074
SLEDAI‐2K score	9.69 (3.46)	7.57 (6.08)	0.429	9.69 (3.46)	10.00 (5.30)	0.070
PGA	1.71 (0.57)	1.35 (0.70)	0.552	1.71 (0.57)	1.60 (0.78)	0.161
Organ involvement^a^						
Mucocutaneous	7 (43.8)	37 (38.9)	0.098	7 (43.8)	16 (51.6)	0.158
Musculoskeletal	4 (25.0)	33 (34.7)	0.214	4 (25.0)	15 (48.4)	0.500
Renal	7 (43.8)	32 (33.7)	0.208	7 (43.8)	14 (45.2)	0.028
Hematologic	5 (31.2)	28 (29.5)	0.039	5 (31.2)	9 (29.0)	0.048
Constitutional	2 (12.5)	16 (16.8)	0.123	2 (12.5)	8 (25.8)	0.343
Immunological	16 (100.0)	63 (66.3)	1.000	16 (100.0)	25 (80.6)	0.693
Vascular	1 (6.2)	4 (4.2)	0.092	1 (6.2)	1 (3.2)	0.143
Serositis	1 (6.2)	8 (8.4)	0.083	1 (6.2)	5 (16.1)	0.317
CNS	0 (0.0)	0 (0.0)	/	0 (0.0)	0 (0.0)	/
Blood test						
Anti‐dsDNA positive	15 (93.8)	53 (68.8)	0.674	15 (93.8)	21 (70.0)	0.648
Low complement C3	11 (68.8)	56 (58.9)	0.205	11 (68.8)	24 (77.4)	0.196
Low complement C4	10 (62.5)	44 (46.3)	0.329	10 (62.5)	21 (67.7)	0.110
24hUP > 0.5 g	5 (33.3)	19 (27.1)	0.135	5 (33.3)	10 (33.3)	<0.001
Medication						
Glucocorticoid^b^	45.00 (10.17)	37.39 (17.36)	0.535	45.00 (10.17)	42.18 (17.71)	0.195
Immunosuppressants						
MMF	10 (62.5)	27 (28.4)	0.728	10 (62.5)	13 (41.9)	0.421
TAC	1 (6.2)	4 (4.2)	0.092	1 (6.2)	1 (3.2)	0.143
CYC	3 (18.8)	28 (29.5)	0.253	3 (18.8)	9 (30.0)	0.264
MTX	1 (6.2)	15 (15.8)	0.308	1 (6.2)	6 (19.4)	0.400
AZA	1 (6.2)	3 (3.2)	0.146	1 (6.2)	1 (3.2)	0.143
CsA	0 (0.0)	10 (10.5)	0.485	0 (0.0)	2 (6.5)	0.371

*Note*. Data were described as mean (SD) or n (percentage).

Abbreviations: ANA, antinuclear antibody; AZA, azathioprine; CNS, central nervous system; CsA, Cyclosporin A; CYC, cyclophosphamide; dsDNA, double‐stranded DNA; MMF, mycophenolate mofetil; MTX, methotrexate; PGA, patient global assessment; SLEDAI, Systemic Lupus Erythematosus Disease Activity Index; TAC, tacrolimus.

^a^ Organ involvement was based on the SLEDAI‐2K affected item.

^b^ The prescribed glucocorticoid dosage was converted to a prednisone equivalent dose.

Among all 47 patients after PSM, 42 (89.4%) patients were female and the mean disease duration was 2.26 (1.42) months. At baseline, the mean (SD) SLEDAI‐2K score was 9.9 (4.7). The most prevalent organ involvement was immunological (87.2%), followed by mucocutaneous (48.9%), renal (44.7%), and musculoskeletal (40.4%) involvement. None of the patients have central nervous system involvement. Hematuria and proteinuria ( > 0.5 g/24 h) were present in 25.6% and 33.3% of patients, respectively. Additionally, estimated glomerular filtration rates (eGFR) less than 90 mL/min and 60 mL/min occurred in 23.3% and 4.7% of patients, respectively. Mycophenolate mofetil was the most commonly used immunosuppressant.

### 3.2 Primary outcome

One patient in the belimumab group achieved LLDAS state at week 12 and sustained it until week 24 (Tables 2 and 3). Among the remaining patients who didn't attain LLDAS at Week 12, 8, of 15 patients reached LLDAS at Week 24. In the control group, two patients achieved LLDAS at both Weeks 12 and 24, while four patients achieved LLDAS until Week 24. A significantly higher proportion of patients in the belimumab group achieved LLDAS at Week 24 compared to the control group (56.3% vs. 19.4%, OR = 5.357, 95%CI = 1.417 to 20.260, *p* = 0.013, Table 3). Given the significant variations in immunosuppressant usage between the two groups after PSM, to mitigate potential confounders, we additionally adjusted for discrepancies in immunosuppressant choice and dosage of GC at baselien as confounding variables in the multivariate logistic analysis. The findings of this analysis reveal that the belimumab group exhibited a significantly higher rate of LLDAS at 24 weeks (OR = 5.143, 95% CI = 1.357 to 19.497, *p* = 0.016, Supporting Information S1: Table S2), which remained statistically significant regardless of the diverse immunosuppressant selections.

**Table 2:** Efficacy results at 12 weeks.

	Belimumab (*n* = 16)	Control (*n *= 31)	OR (95% CI)†	*p*
LLDAS	1 (6.4)	2 (6.5)	0.967 (0.081, 11.544)	.979
Remission	0 (0.0)	1 (3.2)	0.000 (0.000, 0.000)	1.000
Glucocorticoid	15.0 (10.0,16.9)	15.0 (12.5,25.0)	/	.289
≤7.5 mg/d	2 (12.50)	4 (12.9)	0.964 (0.157, 5.928)	.969
Change in SLEDAI‐2K	−6.75 (2.84)	−7.58 (5.26)	/	.486
Reduction ≥4 points in SLEDAI‐2K	14 (87.5)	24 (77.4)	2.042 (0.371, 11.222)	0.412
Change in PGA	−1.15 (0.48)	−0.76 (0.60)	/	0.030
Low complement C3	3 (18.8)	14 (45.2)	0.280 (0.066, 1.184)	0.084
Normalization of low C3	8/11 (72.7)	11/25 (44.0)	3.394 (0.725, 15.897)	0.121
Low complement C4	1 (5.6)	6 (19.4)	0.278 (0.030, 2.536)	0.256
Normalization of low C4	9/10 (90.0)	16/21 (76.2)	2.813 (0.283, 27.971)	0.378
Anti‐dsDNA positive	9 (55.3)	12/22 (54.5)	1.071 (0.293, 3.916)	0.917
Anti‐dsDNA positive to negative	6/15 (40.0)	5/16 (31.3)	1.467 (0.335, 6.430)	0.612

^†^ The ORs was unadjusted.

Abbreviations: SLEDAI, Systemic Lupus Erythematosus Disease Activity Index; PGA, patient global assessment; LLDAS, lupus low disease activity status.

**Table 3:** Efficacy results at 24 weeks.

	Belimumab (*n* = 16)	Control (*n* = 31)	OR(95% CI)^a^	*P*
LLDAS	9 (56.3)	6 (19.4)	5.357 (1.417, 20.260)	0.013
Remission	4 (25.0)	2 (6.5)	4.833 (0.779, 30.005)	0.091
Glucocorticoid	7.5 (5.0,9.4)	10.0 (7.5,12.5)	/	0.008
≤7.5 mg/d	12 (75.0)	11 (35.5)	5.455 (1.414, 21.035)	0.014
Change in SLEDAI‐2K	−7.31 (2.80)	−6.94 (4.22)	/	0.749
Reduction ≥4 points in SLEDAI‐2K	15 (93.8)	23 (74.2)	5.217 (0.591, 46.074)	0.137
Change in PGA	−1.43 (0.61)	−0.92 (0.68)	/	0.017
Low complement C3	3 (18.8)	12/29 (41.4)	0.558 (0.134, 2.317)	0.422
Normalization of low C3	8/11 (72.7)	12/23 (52.2)	2.444 (0.514, 11.619)	0.261
Low complement C4	1 (6.3)	4/29 (13.8)	0.417 (0.042, 4.085)	0.452
Normalization of low C4	9/10 (90.0)	16/19 (84.2)	1.688 (0.152, 18.714)	0.670
Anti‐dsDNA positive	8 (50.0)	11/21 (52.4)	1.212 (0.311, 4.730)	0.782
Anti‐dsDNA positive to negative	7/15 (46.7)	5/16 (31.3)	1.925 (0.445, 8.331)	0.381

^a^ The ORs was unadjusted.

Abbreviations: LLDAS, lupus low disease activity status; PGA, patient global assessment; SLEDAI, Systemic Lupus Erythematosus Disease Activity Index.

### 3.3 Secondary outcomes

In this study, there was no significant difference in the proportion of remission between the belimumab group and the control group at Week 12 or Week 24. However, a higher proportion of patients in the belimumab group had their GC dose reduced to ≤7.5 mg/day at Week 24 compared to the control group (75.0% vs. 35.5%; *p* = 0.001). Similarly, we selected immunosuppressant and baseline GC levels as confounding variables in our analysis. The results of the multivariable analysis remained robust, demonstrating that a significantly higher proportion of patients in the belimumab group achieved a reduction in GC dosage to below 7.5 mg at week 24 (OR = 5.182, 95%CI = 1.339 to 20.058, *p* = 0.017). This finding underscores the potential therapeutic benefit of belimumab in managing GC dosage effectively.

### 3.4 Other outcomes

**Figure 1:**
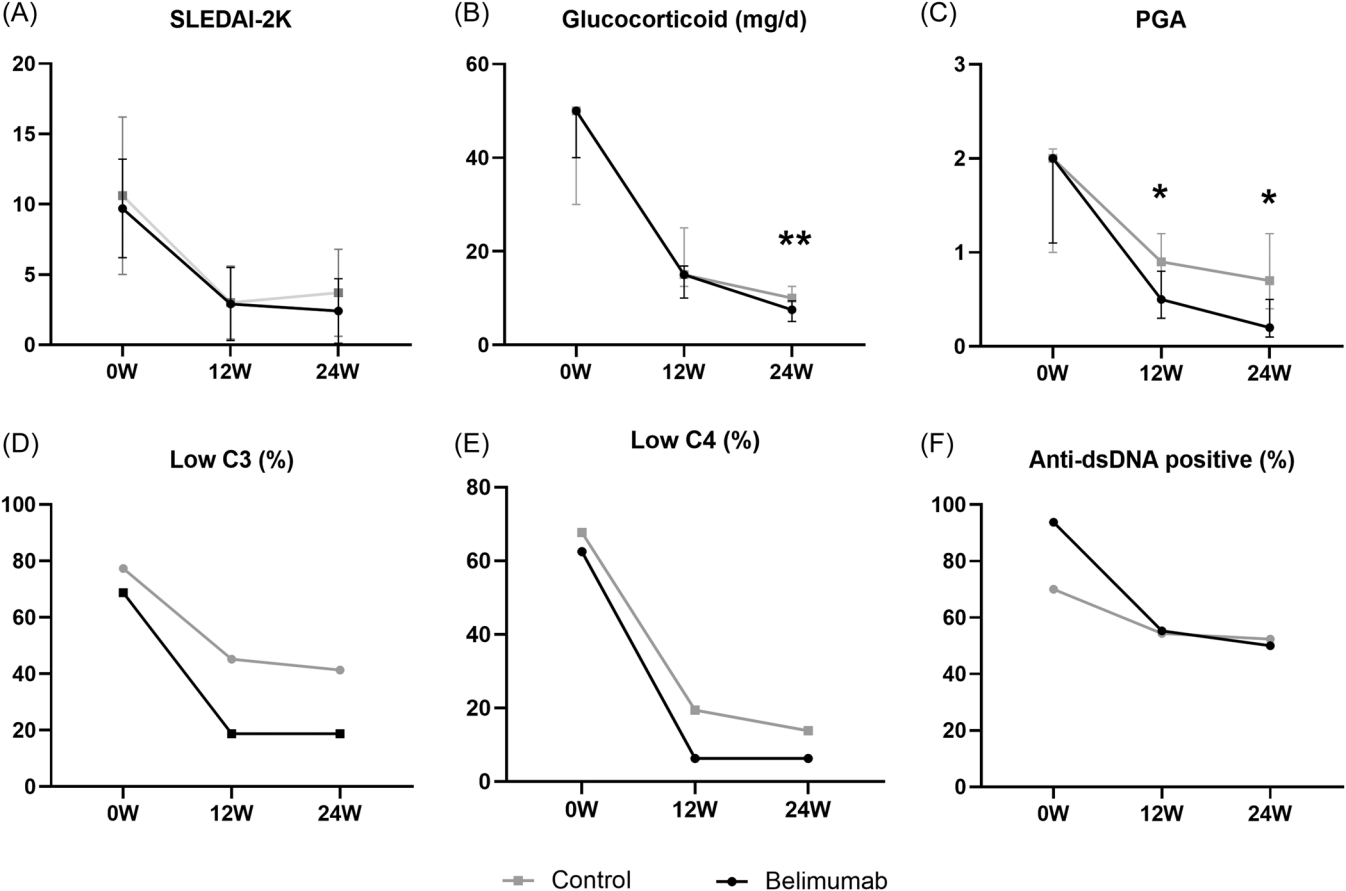
Efficacy results over Time in the belimumab group and control group. *p < .5; **p < .01; (A) SELENA‐2K over 24 weeks. Data are presented as mean (SD); (B) Glucocorticoids dose over 24 weeks. The prescribed glucocorticoid dosage was converted to a prednisone equivalent dose. Data are presented as median (IQR); (C) PGA over 24 weeks. Data are presented as median (IQR); (D) Percentage of low C3 complement concentrations; (E) Percentage of low C4 complement concentrations; (F) Percentage of patients with positive anti‐dsDNA. Abbreviations: dsDNA, double‐stranded DNA; PGA, patient global assessment; SLEDAI, Systemic Lupus Erythematosus Disease Activity Index.

Patients in the belimumab group demonstrated a greater reduction in PGA score than the control group at week 12 (−1.15 vs. −0.76; *p* = 0.03) and Week 24 (−1.43 vs −0.92; *p* = 017). There were no significant differences between the two groups in terms of change in SLEDAI‐2K scores and the proportion of patients achieving an improvement of at least 4 points in the SLEDAI‐2K score. The percentage of normalization of low complement and the conversion of anti‐dsDNA from positive to negative were also similar in both groups at Weeks 12 and 24. At Week 24, the median GC dose in the belimumab group was significantly lower than the control group (7.5 mg/day vs. 10 mg/day, respectively; *p* = 0.008). None of the patients in either group discontinued oral GC therapy after 24 weeks of treatment (shown in Figure 1, Table 2).

### 3.5 Efficacy in lupus nephritis

We observed that the proportion of patients achieving LLDAS at week 24 was slightly higher in those without lupus nephritis (LN) compared to those with LN (42.3% vs 19.0%, *p* = 0.083). Furthermore, the impact of belimumab on early SLE in achieving LLDAS at week 24 was statistically significant only in patients without LN (OR = 11.38; 95% CI = 1.65 to 78.38) (Supporting Information S1: Table S3).

The findings suggest that the efficacy of belimumab may be influenced by renal involvement. Additionally, it is worth noting that the kidney plays a crucial role in SLE. Therefore, we conducted further analysis focusing on patients with LN to assess their renal‐related treatment outcomes (Table 4). The characteristics of patients with LN after PSM at baseline are presented in Supporting Information S1: Table S4. Among the 7 LN patients treated with belimumab, the proportions achieving complete renal response (CRR) at Weeks 12 and 24 were 28.6% and 42.9%, respectively. Two patients transitioned from Nonresponders at week 12 to partial renal response at Week 24. In the control group, although 35.7% of patients achieved complete remission and response (CRR) at Week 12, one patient experienced a flare‐up, resulting in a decrease in the CCR rate to 28.6% at Week 24. A great reduction in urinary protein levels was observed in patients receiving belimumab treatment, with 85.7% of patients achieving a uPCR/24hUP ratio of less than 0.5. In terms of the components of CRR, meeting the criteria for inactive urinary sediment and a uPCR/24hUP ratio below 0.5 proved challenging for the belimumab and control groups respectively. Due to substantial differences in baseline characteristics between the two groups and limited sample size, no further statistical tests were conducted to compare the two groups.

**Table 4:** Efficacy results in lupus nephritis at 12 weeks and 24 weeks.

	12 W		24 W	
	Belimumab (*n* = 7)	Control (*n* = 14)	Belimumab (*n* = 7)	Control (*n* = 14)
CRR	2 (28.6)	5 (35.7)	3 (42.9)	4 (28.6)
PRR	1 (14.3)	1 (7.1)	0 (0.0)	1 (7.1)
NR	4 (57.1)	8 (57.1)	4 (57.1)	9 (64.3)
CRR component				
uPCR/24hU*p* ≤ .5	5 (71.4)	8 (57.1)	6 (85.7)	7 (50.0)
Inactive urinary sediment	4 (57.2)	10 (71.4)	5 (71.4)	11 (78.6)
eGFR not worse than 10% or ≥90 mL/min	6 (85.7)	13 (92.9)	5 (85.7)	11 (78.6)
PRR component				
decrease in the uPCR ≥50%	6 (85.7)	9 (64.3)	6 (85.7)	9 (64.3)
uRBC≥50% reduction or <5 RBC/HPF	4 (57.1)	10 (71.4)	5 (71.4)	11 (78.6)
eGFR not worse than 10% or ≥90 mL/min	6 (85.7)	13 (92.9)	6 (85.7)	13 (92.9)

Abbreviations: 24hUP, 24 h proteinuria; CRR, complete renal response; eGFR, Estimated glomerular filtration rate; NR, Nonresponder; PRR, Partial Renal Response; uPCR, Urinary protein creatinine ratio.

## 4 DISCUSSION

Numerous studies have demonstrated that early introduction of biologic therapies in rheumatoid arthritis (RA) can decrease disease activity and decelerate radiographic progression.^26^, ^27^ Therefore, a window of opportunity for early treatment of RA was accepted. Similarly, the significance of early intervention has also been established in SLE. E Ciruelo et al. found that delaying treatment for more than 5 months was associated with an increased frequency of glomerulonephritis relapses.^28^ Previous clinical trials of belimumab had enrolled patients with a mean disease duration of 5–7 years,^10^, ^29^ and data on the administration of belimumab in early SLE was limited. In this study, we evaluated the clinical efficacy of belimumab in early SLE, defined as a disease duration of fewer than 6 months, and confirmed the efficacy of early introduction of belimumab in SLE.

According to the results of this study, the use of belimumab can aid patients with early SLE in achieving LLDAS. LLDAS is one of the crucial goals in the treat‐to‐target therapeutic strategy,^30^, ^31^ as maintaining LLDAS for at least 50% of the observation period has been associated with a significant reduction in flares.^31^ A cohort study reported that the inability to achieve LLDAS 6 months after treatment initiation is an independent predictor of early damage,^32^ and failure to attain LLDAS was associated with increased mortality.^33^ Our study compared the proportions of patients achieving LLDAS in both groups and demonstrated that individuals receiving belimumab had a higher percentage of LLDAS at week 24, implying that belimumab can effectively manage disease activity in early SLE.

In addition to its disease‐controlling effects, our study has found that belimumab can aid in tapering glucocorticoid dosage for early SLE. GCs are one of the main therapeutic agents for SLE and are among the most useful drugs for inducing rapid remission.^34^ However, prolonged GC exposure is associated with numerous serious complications, including osteoporosis, infections, organ damage, and increased mortality, with the effects being dose‐dependent.^35^ GC doses above 7.5 mg/day were associated with irreversible organ damage.^36^, ^37^, ^38^ It is worth noting that GC doses administered during the early stages of SLE can influence long‐term prognosis.^39^ Therefore, the 2019 European League Against Rheumatism (EULAR) guidelines recommend that GCs should be minimized to less than 7.5 mg/day dosage of prednisone equivalent in maintaining treatment.^40^ Our findings, consistent with prior research on belimumab,^38^, ^41^ suggest that belimumab has a superior impact on decreasing GC and improving prognoses, even when administered to individuals with early‐stage SLE.

Kidneys are frequently affected in SLE, making them a crucial organ of concern in disease management. The BLISS‐LN study assessed the efficacy of belimumab on LN and demonstrated significant improvements in outcomes.^11^ In addition, a previous observational cohort study conducted in China demonstrated the effectiveness and safety of belimumab for treating Lupus nephritis in real‐world clinical practice.^42^ However, the study population had a median disease duration ranging from 3.3 to 8 years. To address this knowledge gap, our study aimed to evaluate the efficacy of belimumab treatment in patients with early‐stage LN. Following belimumab therapy, all SLE patients demonstrated a significant reduction in urinary protein levels and stable creatinine levels, with some achieving complete remission. These findings provide further evidence supporting the favorable impact of belimumab on renal involvement. The longitudinal analysis of CRR outcomes in the two groups suggests that the addition of belimumab may contribute to maintaining disease remission and preventing relapse, compared to conventional standard of care. The previous studies indicate that patients with LN treated with belimumab achieve their highest CRR at approximately 52 weeks,^11^ suggesting that a more extended follow‐up period is necessary to evaluate the efficacy of belimumab in early‐stage LN patients.

We must acknowledge some limitations of this study. Firstly, it is important to note that this study was conducted retrospectively. Despite our efforts to mitigate bias through the use of propensity score matching (PSM) and inclusion of objective data, some imbalance between the two groups was still observed, such as disparities in the selection of immunosuppressive agents. Although logistic regression analysis was employed to address potential confounding factors, there remains a possibility of overestimating or underestimating the efficacy of belimumab. Additionally, the sample size of this study was relatively small and may require support results from larger sample sizes in the future. Finally, as a retrospective study, some data is difficult to obtain, such as adverse events about treatment.

## 5 CONCLUSION

In conclusion, our study suggests that belimumab contributed to reaching LLDAS status and reducing GC doses in SLE patients with a disease duration of fewer than 6 months, which was defined as early SLE. We have reason to believe that the use of belimumab in patients at an early stage is reasonable, especially in patients with long‐term contraindications to steroid use and a high risk of associated complications.

## AUTHOR CONTRIBUTIONS

Xiaomei Leng and Xiaofeng Zeng contributed to the study's conception and design; all authors contributed to material preparation, and data collection; Chaofan Lu contributed to data analysis. The first draft of the manuscript was written by Chaofan Lu and all authors commented on previous versions of the manuscript.

## CONFLICT OF INTEREST STATEMENT

The authors declare no conflict of interest.

## ETHICS STATEMENT

This study was reviewed and approved by the Medical Ethics Committee of Peking Union Medical College Hospital and was in accordance with the Declaration of Helsinki, approval number (ZS‐3063). All patients gave their signed informed consent. Observational data were collected, pseudonymized, and analyzed by group rather than on an individual basis, without exposing individual patients.

## Supporting information

Supporting information.

## Data Availability

All data generated or analyzed during this study are included in this article. Further enquiries can be directed to the corresponding author.
