# Supplementary material for: Belimumab in early systemic lupus erythematosus: A propensity score matching analysis
Source: Immun Inflamm Dis. 2024 Aug 22;12(8):e1362. doi: 10.1002/iid3.1362 (PMC11340010; doi:10.1002/iid3.1362)
Supplement: Supplementary file 1 — Supporting information. [file IID3-12-e1362-s001.docx]

**Belimumab in early systemic lupus erythematosus: a propensity score matching analysis**

**Journal: Immunity, Inflammation and Disease**

**Authors**: Chaofan Lu, Nan He, Lei Dou , Hongxia Yu, Mengtao Li, Xiaomei Leng, Xiaofeng Zeng

**Corresponding Author:** Xiaofeng Zeng and Xiaomei Leng

**Email**: [zengxfpumc@163.com](mailto:zengxfpumc@163.com); [lpumch@126.com](mailto:lpumch@126.com).

**This PDF file includes:**

Supplementary Table s (Table. S1 to S4)

Table. S1 Subgroup analysis for comparing the rate of LLDAS in early SLE based on renal involvement

|  | Belimumab group | Control group | OR (95% CI) | P |
| --- | --- | --- | --- | --- |
| **All patients** | 16 | 31 | 5.36 (1.42, 20.26) | 0.013 |
| Patients without LN | 9 | 17 | 11.38 (1.65, 78.38) | 0.014 |
| Patients with LN | 7 | 14 | 2.40 (0.26, 22.11) | 0.440 |

Abbreviations: LN, lupus nephritis; LLDAS, LLDAS, lupus low disease activity status.

Table. S2 Demographic and clinical characteristics of patients with LN after PSM at baseline

|  |  | | |
| --- | --- | --- | --- |
|  | Control  (n=14) | Belimumab (n=7) | SMD |
| Gender, female | 14 (100.0) | 7 (100.0) | <0.001 |
| Age, years | 35.43 (9.43) | 33.71 (13.14) | 0.150 |
| Disease duration, months | 1.93 (1.00) | 2.43 (1.40) | 0.412 |
| SLEDAI-2K score | 13.14 (3.80) | 11.71 (3.68) | 0.382 |
| PGA | 1.76 (0.80) | 1.93 (0.45) | 0.252 |
| Organ involvement † |  |  |  |
| Mucocutaneous | 5 (35.7) | 2 (28.6) | 0.153 |
| Musculoskeletal | 6 (42.9) | 1 (14.3) | 0.667 |
| Hematologic | 7 (50.0) | 2 (28.6) | 0.450 |
| Constitutional | 2 (14.3) | 0 (0.0) | 0.577 |
| Immunological | 13 (92.9) | 7 (100.0) | 0.392 |
| Vascular | 0 (0.0) | 1 (0.0) | / |
| Serositis | 2 (14.3) | 0 (0.0) | 0.577 |
| CNS | 0 (0.0) | 0 (0.0) | / |
| Blood test |  |  |  |
| Anti-dsDNA positive | 12 (85.7) | 6 (85.7) | <0.001 |
| Low complement C3 | 1 (7.1) | 1 (14.3) | 0.232 |
| Low complement C4 | 2 (14.3) | 2 (28.6) | 0.354 |
| 24hUP>0.5g | 10 (76.9) | 5 (71.4) | 0.126 |
| Medication |  |  |  |
| Glucocorticoid‡ | 50.71 (10.72) | 47.86 (9.06) | 0.288 |
| Immunosuppressants |  |  |  |
| MMF | 5 (35.7) | 4 (57.1) | 0.440 |
| TAC | 1 (7.1) | 1 (14.3) | 0.232 |
| CYC | 6 (46.2) | 2 (28.6) | 0.370 |
| MTX | 0 (0.0) | 0 (0.0) | / |
| AZA | 1 (7.1) | 0 (0.0) | 0.392 |
| CsA | 2 (14.3) | 0 (0.0) | 0.577 |

Data were described as mean (SD) or n (percentage).

† Organ involvement was based on the SLEDAI-2K affected item.

‡ The prescribed glucocorticoid dosage was converted to a prednisone equivalent dose.

Abbreviations: SLEDAI, Systemic Lupus Erythematosus Disease Activity Index; PGA, patient global assessment; CNS, central nervous system; ANA, antinuclear antibody; dsDNA, double-stranded DNA; MMF, mycophenolate mofetil; TAC, tacrolimus; CYC, cyclophosphamide; MTX, methotrexate; AZA, azathioprine; CsA, Cyclosporin A.

Table. S3 Efficacy results at 12 weeks after adjustment for baseline treatment

|  | Belimumab  (n=16) | Control  (n=31) | adjusted OR (95% CI) | adjusted P |
| --- | --- | --- | --- | --- |
| LLDAS | 1 (6.4) | 2 (6.5) | 1.423 (0.076, 26.671) | 0.813 |
| Remission | 0 (0.0) | 1 (3.2) | 0.000 (0.000, 0.000) | 1.000 |
| Glucocorticoid | 15.0  (10.0,16.9) | 15.0 (12.5,25.0) | / | 0.040 |
| GC ≤7.5mg/d | 2 (12.50) | 4 (12.9) | 0.615 (0.065, 5.866) | 0.673 |
| Reduction ≥4 points in  SLEDAI-2K | 14 (87.5) | 24 (77.4) | 1.876 (0.261, 13.488) | 0.532 |
| Change in PGA | -1.15 (0.48) | -0.76 (0.60) | / | 0.009 |
| Low complement C3 | 3 (18.8) | 14 (45.2) | 0.242 (0.049, 1.196) | 0.082 |
| Normalization of low C3 | 8/11 (72.7) | 11/25 (44.0) | 4.719 (0.696, 31.993) | 0.112 |
| Low complement C4 | 1 (5.6) | 6 (19.4) | 0.234 (0.023, 2.379) | 0.219 |
| Normalization of low C4 | 9/10 (90.0) | 16/21 (76.2) | 2.859 (0.255, 32.085) | 0.395 |
| Anti-dsDNA positive | 9 (55.3) | 12/22 (54.5) | 1.886 (0.371, 9.590) | 0.445 |
| Anti-dsDNA positive to negative | 6/15 (40.0) | 5/16 (31.3) | 13.835 (0.754, 253.689) | 0.077 |

Abbreviations: SLEDAI, Systemic Lupus Erythematosus Disease Activity Index; PGA, patient global assessment; LLDAS, lupus low disease activity status.

Table. S4 Efficacy results at 24 weeks after adjustment for baseline treatment

|  | Belimumab (n=16) | Control  (n=31) | adjusted OR (95% CI) | adjusted P |
| --- | --- | --- | --- | --- |
| LLDAS | 9 (56.3) | 6 (19.4) | 5.143 (1.357, 19.497) | 0.016 |
| Remission | 4 (25.0) | 2 (6.5) | 4.663 (0.567, 38.374) | 0.152 |
| Glucocorticoid | 7.5  (5.0,9.4) | 10.0  (7.5,12.5) | / | 0.016 |
| ≤7.5mg/d | 12 (75.0) | 11 (35.5) | 5.182 (1.339, 20.058) | 0.017 |
| Change in SLEDAI-2K | -7.31 (2.80) | -6.94 (4.22) | / | 0.718 |
| Reduction ≥4 points in SLEDAI-2K | 15 (93.8) | 23 (74.2) | 6.242 ( 0.418, 93.134) | 0.184 |
| Change in PGA | -1.43 (0.61) | -0.92 (0.68) | / | 0.009 |
| Low complement C3 | 3 (18.8) | 12/29 (41.4) | 0.242( 0.047, 1.255) | 0.091 |
| Normalization of low C3 | 8/11 (72.7) | 12/23 (52.2) | 3.137 ( 0.498, 19.751) | 0.223 |
| Low complement C4 | 1 (6.3) | 4/29 (13.8) | 0.398(0.037, 4.319) | 0.449 |
| Normalization of low C4 | 9/10 (90.0) | 16/19 (84.2) | 2.152 ( 0.172, 26.885) | 0.552 |
| Anti-dsDNA positive | 8 (50.0) | 11/21 (52.4) | 1.839 (0.399, 8.473) | 0.434 |
| Anti-dsDNA positive to negative | 7/15 (46.7) | 5/16 (31.3) | 4.412 (0.675, 28.856) | 0.121 |

Abbreviations: SLEDAI, Systemic Lupus Erythematosus Disease Activity Index; PGA, patient global assessment; LLDAS, lupus low disease activity status.
